# The impact of radiation dose on the efficacy of definitive chemoradiotherapy in patients with locally advanced esophageal carcinoma: a systematic review and meta-analysis

**DOI:** 10.1080/15384047.2022.2156246

**Published:** 2022-12-15

**Authors:** Danjing Luo, Qiulu Zhong, Xiaodong Zhu

**Affiliations:** a Department of Radiation Oncology, Guangxi Medical University Cancer Hospital, Nanning, P.R.China; b Department of Radiation Oncology, Second Affiliated Hospital of Guangxi Medical University, Nanning, China; c Department of Oncology, Wuming Hospital of Guangxi Medical University, Nanning, China

**Keywords:** High-dose, low-dose, chemoradiotherapy, locally advanced esophageal carcinoma, Meta-analysis

## Abstract

To investigate the impact of radiation dose on the efficacy of definitive chemoradiotherapy(dCCRT) in patients with locally advanced esophageal carcinoma. PubMed, EMBASE, Cochrane Central Register of Controlled Trials, Wanfang, and Chinese National Knowledge Infrastructure(CNKI) were searched for eligible studies. Studies that compared high-dose radiation(HD-RT) group with low-dose radiation(LD-RT) group using modern radiotherapy techniques for locally advanced esophageal carcinoma patients in dCCRT were identified. The hazard ratios (HR) for overall survival (OS), progression-free survival (PFS), and the odds ratios (OR) for clinical complete response (cCR), local–regional failure (LRF), distant metastasis (DM), and grade≥3 AEs. Meta-analysis was performed when relevant data were available. Eleven studies involving 1943 patients were included for analyses. The results showed that the HD-RT group had better OS (pooled HR 0.78 [0.70, 0.87], p < .00001), PFS (pooled HR 0.72 [0.55, 0.94], p = .01), cCR (OR 1.52 [1.13, 2.05], p = .005), and LRF (OR 0.60 [0.45, 0.80], p = .0004). In addition, there were no significant differences between the two groups in terms of DM (OR 1.43 [1.00, 2.04], p = .05), grade 3–5 radiation pneumonitis (OR 1.38 [0.71, 2.68], p = .35), grade 3–5 radiation esophagitis (OR 1.36 [0.88, 2.10], p = .17), grade 3–5 other esophageal toxicities(stenosis/fistula/hemorrhage) (OR 1.22 [0.75, 2.00], p = .43), and treatment-related death (OR 1.40 [0.73, 2.68], p = .31). High-dose radiotherapy in definitive CCRT for patients with locally advanced esophageal carcinoma is associated with improved PFS, OS, cCR, and LC with no increase of grade≥3AEs. Simultaneously, we await the preliminary and final results of several ongoing dose-escalation randomized trials. Furthermore, future studies should provide personalized radiotherapy doses for these patients.

## Introduction

Patients with locally advanced esophageal carcinoma (LAEC) account for approximately 50% of the total,^1^ and the majority of them have lost the opportunity for surgery at the time of diagnosis. The currently recommended treatment modality for these unresectable patients is platinum-based definitive concurrent chemoradiotherapy (CCRT) based on the result of the Intergroup Radiation Therapy Oncology Group (RTOG)- 8501.^2^ The recommended dose by National Comprehensive Cancer Network (NCCN) was 50–50.4 Gy^3^ based on the results of Intergroup Radiation Therapy Oncology Group (RTOG)90–12^4^and INT-0123 (also known as RTOG 94–05).^5^ Although even with this modality therapy, survival remains disappointing and with 5-year overall survival (OS) rate of approximately 20%, and the most common mode of treatment failure is locoregional recurrence within the gross tumor volume, which was as high as 50%,^6^ especially in patients with LAEC.^7,8^ Simultaneously, dose escalation has been shown in numerous clinical trials and meta-analyses to improve local control (LC) and OS with no increase in serious side effects, raising the possibility that this factor may be advantageous in CCRT.^9–13^ Nevertheless, the ARTDECO study^14^ and the study undertaken by Xu et al^15^came to oppose, indicating that dose escalation has no benefit on OS. Hence, the recommended radiation dose remains controversial. However, many studies included patients at all stages, which may have impacted the findings and reduced the effect of current clinical evidence. Hence, we performed this up-to-date meta-analysis to determine whether dose escalation of CCRT could improve the survival of patients with LAEC.

## Patients and methods

### Search strategy

This study was registered on the PROSPERO database (number CRD42022330871). The following keywords were used to search PubMed, EMBASE, Cochrane Central Register of Controlled Trials, Wanfang, and Chinese National Knowledge Infrastructure (CNKI) for literature published before June 2022:

((“esophageal” [Title]) or (“oesophageal” [Title]) or (“esophagus” [Title])) and ((“tumor” [Title]) or (“cancer” [Title]) or (“carcinoma” [Title]) or (“neoplasm” [Title]) or (“neoplasms” [Title])) and ((“chemoradiation” [Title]) or (“chemoradiotherapy” [Title]) or (“radiochemotherapy” [Title]) or (“chemo-irradiation” [Title]) or (“chemo-radiotherapy” [Title]))and ((“dose”[Abstract]) or(“dose escalation”

[Abstract]) or(“dose-escalated”[Abstract]) or(“high dose”[Abstract])). Manual searches of reference lists were also performed.

### Study election

Inclusion criteria included: 1) Studies on patients with LAEC (AJCC 6^th^: stage II–IVA; AJCC 7^th^: stage IB-IIIC; AJCC 8^th^: stage IB-IVA). 2) Studies comparing the curative efficacy in LAEC patients with HD-RT or LD-RT. 3) OS must be reported. 4)The most recent and informative publication from the same trial. 5)The language limit to English and Chinese.

The following studies were excluded: 1) Studies on patients with distant metastasis or with other cancers.2) Only 2D radiotherapy techniques or Co-60. 3) single-arm trial, letters, review, case report, meta-analysis, or abstract of meeting.

### Data extraction

The following information was gathered from all included studies:1) information and characteristics: first name of author, nation, year of publication, study period, sort of research, clinical stage, gender, histology, groups, patient number, location, radiation dose, regimens, radiation technology, quality.2) primary data: HR and 95% CI of OS and PFS; incidence rate for cCR, LRF, and DM, and grade≥3 adverse events(AEs). Engauge Digitizer version 4.1 (available from: http://digitizer.sourceforge.net/) was applied to read the survival rates from Kaplan-Meier curves, and then the spreadsheet attached to Tierney’s paper was used to calculate HR.

### Quality assessment

The quality of all included studies was rated separately by two evaluators. The 9-star Newcastle-Ottawa Scale (Available from: http://www.ohri.ca/programs/clinical_epidemiology/oxford.htm) was used to assess non-RCTs, with high quality scoring 7–9, medium quality scoring 4–6, and low quality scoring 1–3. The 7-point JADAD scale was used to assess RCTs, with high quality scoring 4–7 and poor quality scoring 1–3.

### Statistical analysis

This meta-analysis was carried out using the software of the Review Manager (Rev Man) (version 5.3) and STATA v12.0. I^2^ was used to assess statistical heterogeneity. If I^2^ ≤ 50%, a fixed-effects model was conducted to synthesize HR and OR; otherwise, a random-effects model was used. The tests were considered statistically significant if P < .05. All the P values were two-sided. Begg’s and Egger’s tests were used to examine the publication bias of PFS and OS. Sensitivity analysis was used to determine the effect of any individual study on the final results.

## Results

**Figure 1.**
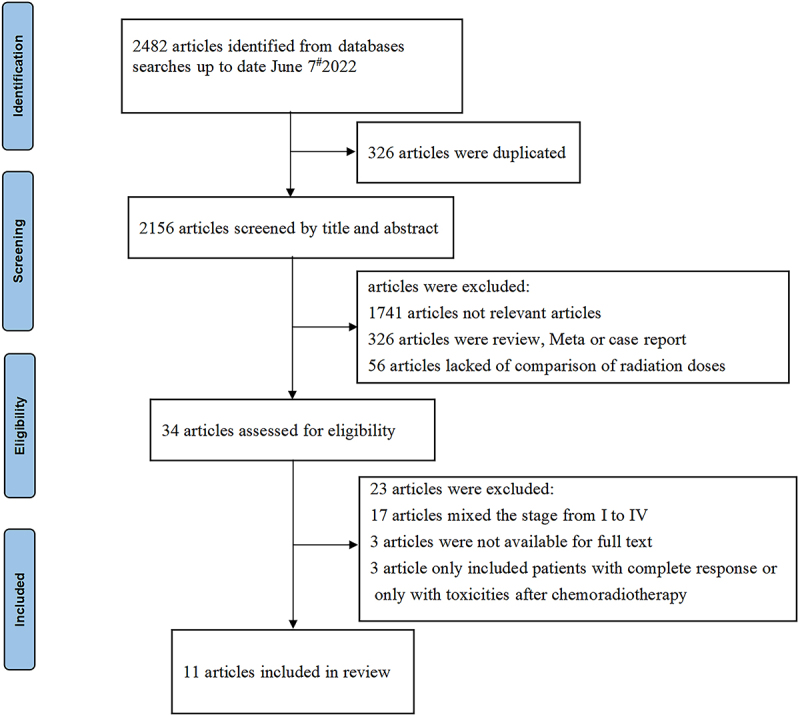
Flow chart of studies selection procedure.

Eleven studies met the criteria and were incorporated into the meta-analysis (Figure 1 outlines the selection process flow). The eleven studies consisted of four randomized controlled trials (RCTs), three population-based propensity-score-matched analyses, and four retrospective studies. There was a total of 1943 LAEC patients, of whom 962 received LD-RT while 981 received HD-RT. The detailed information of all studies is reported in Table 1.^14–24^

**Table 1.** Basic characteristics of the included studies.

Author	Nation	Year	Study period	Study design	Clinical stage	Gender (M/F)	Histology types (SCC /Other)	Groups	Patients number	Location(Cervical/ upper/middle/lower/other)	Radiation Dose groups	Chemotherapy regimens	Radiation technology	Studies Quality
Zhu^16^	China	2012	1/2007-12/2007	prospective	II–III (AJCC6^th^)	-	44/0	Low dose High dose	24 20	44(c + u)/0/0/0	60 Gy 63.9 Gy (SIB)	PF	IMRT	5
Suh^17^	Korea	2014	1/1998-2/2008	retrospective	II–III (AJCC6^th^)	117/9	117/9	Low dose High dose	49 77	1/10/24/14/0 9/14/41/13/0	54 Gy (45–59.4) 63 Gy (60–75.6)	PF/5-Fu	3D-CRT	7
Chen^18^	China, Taiwan	2016	2008–2013	retrospective	II–IV (AJCC6/7^th^)	619/29	648/0	Low dose High dose	324 324	-	50–50.4 Gy ≥60 Gy	CCRT	3D-CRT, IMRT	5
Kim^19^	Korea	2017	1994–2013	retrospective	II–III (AJCC6/7^th^)	226/10	230/6	Low dose High dose	120 116	5/27/55/33/0 11/31/58/16/0	50.4 Gy (45–59.4) 63 Gy (60–66.6)	PF /other	3D-CRT, IMRT	6
Nayan ^20^	India	2018	-	Prospective	IIB-IIIB (AJCC7^th^)	18/10	28/0	Low dose High dose	14 14	-	50.4 Gy 64.8 Gy	PF	3D-CRT, IMRT	5
Zhang^21^	China	2018	2010–2014	retrospective	II–III (AJCC7^th^)	47/33	80/0	Low dose High dose	43 37	0/8/16/19/0 0/6/15/16/0	52 Gy (50.4–56) 62 Gy (59.4–64.8)	TP /PF /PS	3D-CRT, IMRT	6
Li^22^	China, Taiwan	2019	2011–2015	retrospective	II–III (AJCC7^th^)	36/0	36/0	Low dose High dose	18 18	-	50 Gy (47.5–52.5) 60 Gy (57–63)	CCRT	IMRT, IGRT	6
Li^23^	China, Taiwan	2021	2011–2017	retrospective	II–III (AJCC7^th^)	52/2	54/0	Standard dose High dose	27 27	27/0/0/0 27/0/0/0	50 Gy 60–70 Gy	CCRT	IMRT, IGRT	6
Hulshof^14^	Netherlands	2021	9/2012-6/2018	Prospective (Phase III)	II–IVA (AJCC7^th^)	179/78	159/98	Low dose High dose	130 130	9/33/27/50/0 4/27/40/50/0	50.4 Gy 61.6 Gy (SIB)	TC	3D-CRT	5
Xu^15^	China	2022	10/5/2013 – 16/5/2017	Prospective (Phase III)	IIA‒IVA (AJCC6^th^)	251/68	319/0	Low dose High dose	159 160	96(c + U)/63(M + L)/0 89(c + U)/71(M + L)/0	50 Gy 60 Gy	DP	IMRT, IGRT	5
Zhu^24^	China	2022	1/2015–12/2019	retrospective	II–III (AJCC7^th^)	85/27	112/0	Low dose High dose	54 58	0/15/25/14 0/19/27/12	50.4 Gy 60 Gy	NS ND	IMRT	7

M Male, F Female, SCC Squamous cell cancer, 3D-CRT Three dimensional conformal radiotherapy, IMRT Intensity-modulated radiotherapy, IGRT Imaging Guided radiation therapy, CCRT Concurrent chemo-radiotherapy, PF Cisplatin + 5-fluorouracil, DCF docetaxel+cisplatin+ 5-fluorouracil,TP cisplatin +paclitaxel,PS Cisplatin +S-1,DP docetaxel + cisplatin,NS Nedaplatin+S-1,ND Nedaplatin+ docetaxel. AJCC 6/7^th^ American Joint Committee on Cancer staging 6/7^th^.

**Figure 2.**
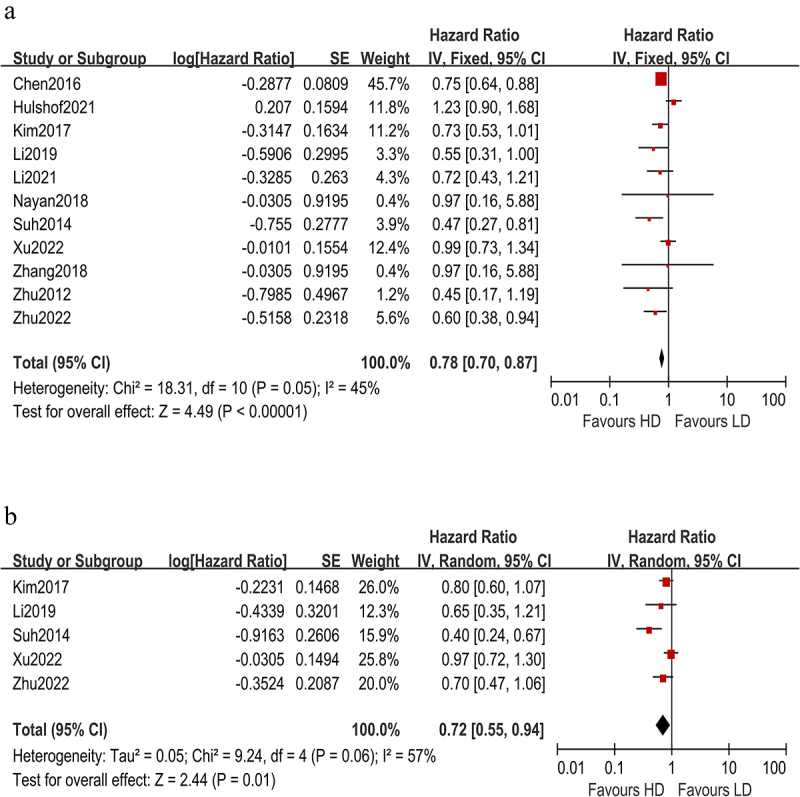
Forest plots for relationship between HD-RT and LD-RT;(a) pooled analyses for OS;(b)pooled analyses for PFS.

All articles reported overall survival in groups. Patients in the HD-RT group had significant survival benefits compared to patients in the LD-RT group (pooled HR 0.78 [0.70, 0.87], p < .00001, Figure 2a). A fixed model was employed because I^2^ < 50% (I^2^ = 45%).

Five studies analyzed the PFS of the two groups. HD-RT group had a significant advantage over LD-RT group (HR 0.72 [0.55, 0.94], p = .01, Figure 2b). A random model was employed because I^2^ > 50% (I^2^ = 57%).

**Figure 3.**
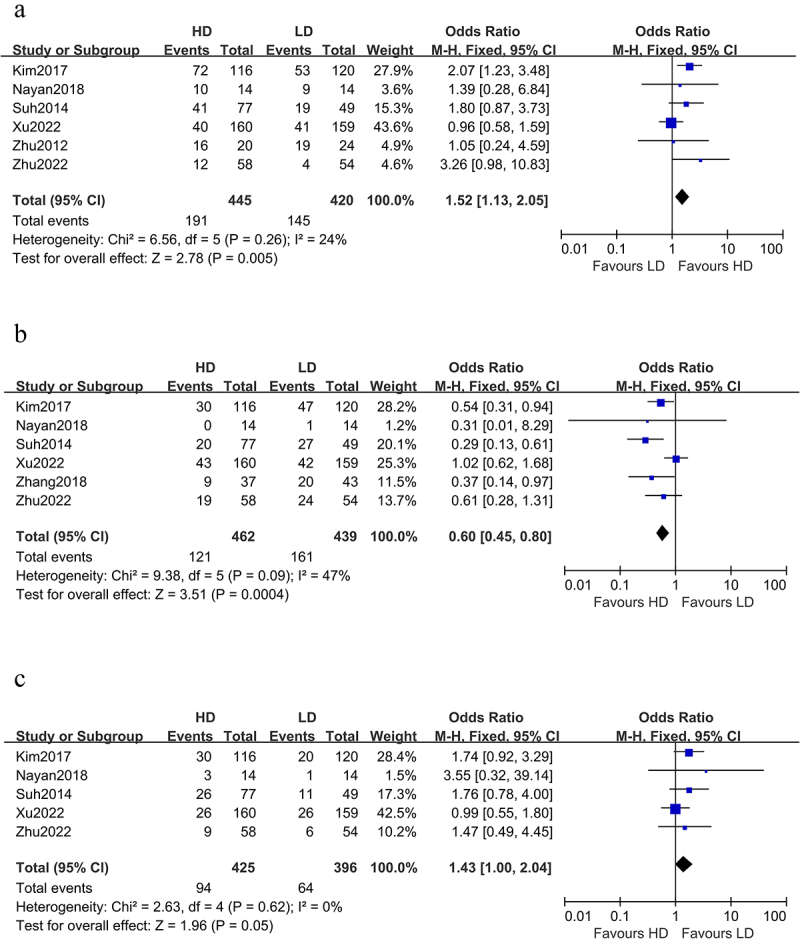
Forest plot for relationship between HD-RT and LD-RT.(a) odds ratio for cCR;(b) odds ratio for LRF;(c) odds ratio for DM.

Six studies compared the cCR and LRF rates of the two groups, with the HD-RT group outperforming the LD-RT group in both cCR (OR 1.52 [1.13, 2.05]; P = .005, Figure 3a) and LRF (OR 0.60 [0.45, 0.80], p = .0004, Figure 3b). A fixed model was employed because I^2^ < 50%.

Five articles analyzed the DM rates of the two groups. There was no difference between the two groups in this respect (OR 1.43 [1.00, 2.04]; P = .05, Figure 3c). A fixed model was employed because I^2^ < 50%.

**Figure 4.**
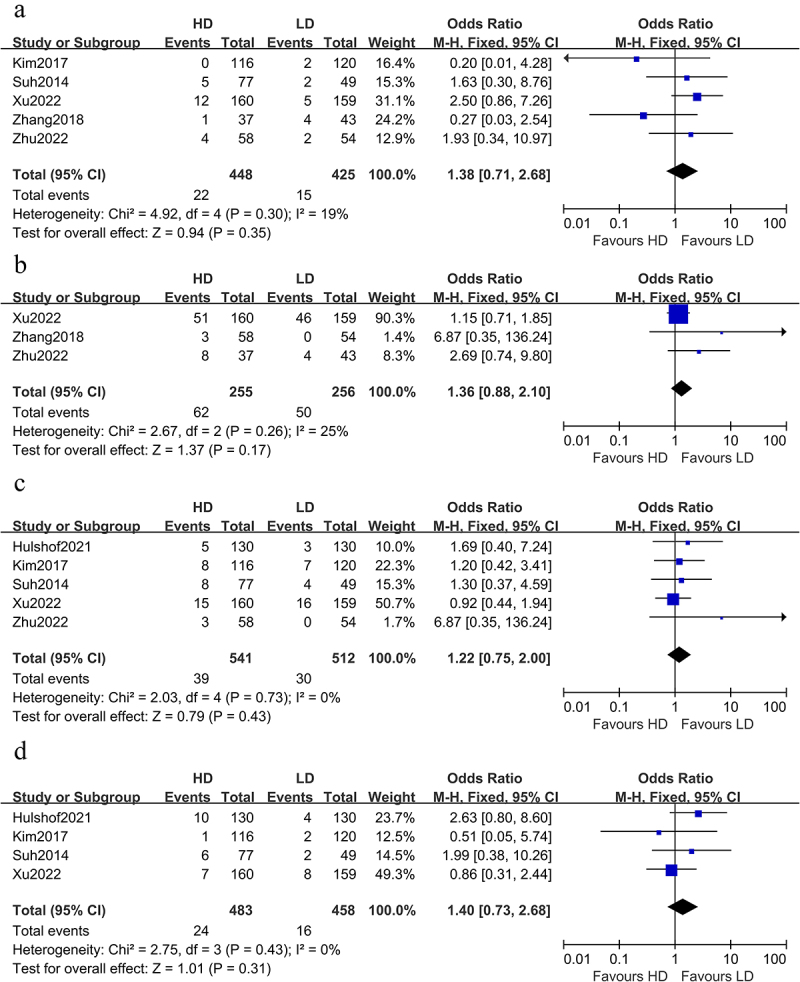
Effect of HD-RT and LD-RT on grade≥3 AEs.Odds ratio for (a)pneumonitis; (b)esophagitis;(c)other esophageal toxicities: stenosis/fistula/hemorrhage;(d)treatment-related death.

Eight articles reported grade ≥ 3AEs (Table 2). No significant difference was demonstrated between the two arms in terms of grade 3–5 radiation pneumonitis (OR 1.38 [0.71, 2.68], p = .35, Figure 4a), grade 3–5 radiation esophagitis (OR 1.36 [0.88, 2.10], p = .17, Figure 4b), grade 3–5 radiation other esophageal toxicities (stenosis/ fistula/ hemorrhage) (OR 1.22 [0.75, 2.00], p = .43, Figure 4c), treatment-related death (OR 1.40 [0.73, 2.68], p = .31, Figure 4d). A fixed model was employed because I^2^ < 50%.

**Table 2.** Adverse events of grades 3–5.

Studies	Patients Number (LD/HD)	Radiation technology	Radiotherapy dose		Pneumonitis	Esophagitis	Other Esophageal toxicities (stenosis/fistula/hemorrhage)	Treatment- Related Death	Evaluation Criterion for toxicities
			LD-RT	HD-RT					
Zhu^16^	24/20	IMRT	60 Gy/2 Gy	63.9 Gy/2.13 Gy	0 vs 0	0 vs 0	0 vs 0	0 vs 0	CTCAE 3.0
Suh^17^	49/77	2D/ 3D-CRT	45–59.4 Gy/1.8–2 Gy	60–75.6 Gy/1.8–2 Gy	4% vs 6%	-	8.2% vs 10.4%	4% vs 7.8%	CTCAE 3.0
Kim^19^	120/116	3D-CRT, IMRT	45–59.4 Gy/1.8–2 Gy	60–66.6 Gy/1.8–2 Gy	1.7% vs 0	-	5.8% vs 6.9%	1.7% vs0.9%	CTCAE 4.0
Nayan^20^	14/14	3D-CRT, IMRT	50.4 Gy/1.8 Gy	64.8 Gy/1.8 Gy	0 vs 0	0 vs 0	0 vs 0	0 vs 0	CTCAE 4.0
Zhang^21^	43/37	3D-CRT, IMRT	50.4–56 Gy/1.8–2 Gy	59.4–64.8 Gy/1.8–2 Gy	9.3%vs27%	9.3% vs 21.6%	-	0 vs 0	CTCAE 4.0
Hulshof^14^	130/130	3D-CRT	50.4 Gy/1.8 Gy	61.6 Gy/2.2 Gy	-	-	2.3% vs 3.8%	3.3% vs7.6%	CTCAE 4.0
Xu^15^	159/160	IMRT, IGRT	50 Gy/2 Gy	60 Gy/2 Gy	3.1% vs 7.5%	28.8%vs31.7%	9.9% vs 9.3%	5.0% vs 4.4%	CTCAE 4.0
Zhu^24^	54/58	IMRT	50.4Gy/1.8 Gy	60 Gy/2.0 Gy	1.9%vs6.9%	0 vs 5.2%	0 vs 5.1%	0 vs 0	CTCAE 4.0

RT radiotherapy, IMRT Intensity-modulated radiotherapy, 3D-CRT Three dimensional conformal radiotherapy, IGRT Imaging Guided radiation therapy, LD low dose, HD high dose;CTCAE Common Terminology Criteria for Adverse Events.

### Sensitivity analysis

**Figure 5.**
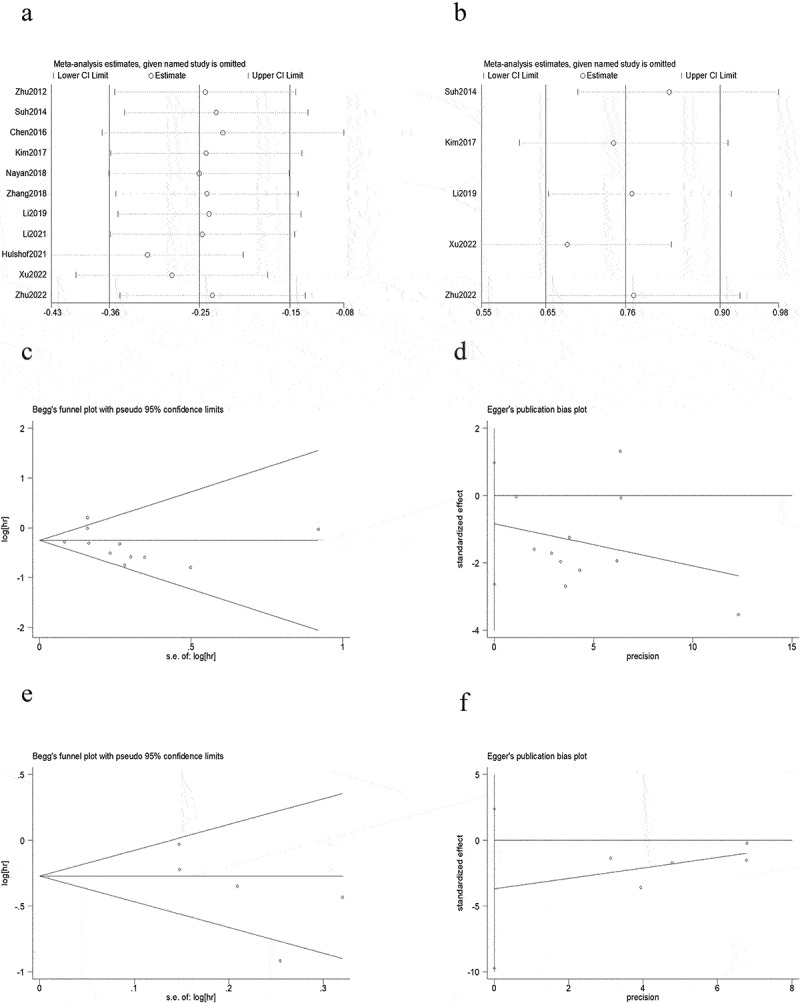
Sensitivity analyses for (a) OS and (b) PFS.Publication bias funnel plot: Beggs test and Eggers test for (c and d) OS and (e and f) PFS.

Sensitivity analysis revealed that the new HRs for OS (Figure 5a) and PFS (Figure 5b) were identical to the original HRs, demonstrating that no single study may have significantly influenced the meta-analysis results.

### Publication bias

No significant publication bias was found between HD-RT group and LD-RT group among all studies on OS (Figure 5c, Begg’s test, P = .533; Figure 5d, Egger’s test, p = .325[−2.6350,0.9731]) and PFS (Figure 5e, Begg’s test, P = .086;Figure 5f, Egger’s test, p = .148[−9.7395, 2.3638]), all P-values were >0.05.

## Discussion

Does dose escalation of CCRT improve the survival of patients with LAEC? In our study, we focus on patients with locally advanced esophageal carcinoma and summarize the current clinical evidence of dose escalation. The findings demonstrated a higher dose utilizing modern radiation techniques for definitive CCRT of LAEC might reduce LRF and improve PFS, OS, and cCR of patients without increasing toxicity rates compared to low-dose radiotherapy. There have been numerous studies on esophageal cancer dose escalation, but no conclusion has been reached. Based on the findings of the INT0123 study,^5^ the dose recommendation for definitive radiotherapy is 50–50.4 Gy. Minsky et al compared the high radiation dose group (64.8 Gy) to the low-dose group (50.4 Gy) with conventional radiotherapy techniques(2D-RT), and no benefit in local control rate or survival rate was observed, but treatment-related mortality increased. It was worth noting that the enrolled population contains a higher proportion of patients in the early stages (I–IIB). Moreover, only the primary tumor was treated up to 64.8 Gy, with no dose escalation for involved nodes. Furthermore, HD group radiotherapy treated patients with squamous cell carcinoma (85.8%) and adenocarcinoma (14.2%). In addition, the radiation dose did not meet 50.4 Gy in 7 of the 11 treatment-related deaths in the high-dose group. As a result, the study spurred controversy in its aftermath. Similar results were in other studies even with modern radiotherapy techniques(3D-RT).^25,26^ Brower et al^25^conducted a population-based retrospective analysis of 6854 patients from the National Cancer Data Base (NCDB) of America, of which 3821 received doses of 50 to 50.4 Gy and 3033 received doses higher than 50.4 Gy, and three matched groups were generated for the comparisons of 50 to 50.4 Gy versus 51 to 54 Gy, 50 to 50.4 Gy versus 55 to 60 Gy, and 50 to 50.4 Gy versus >60 Gy, there were no survival differences between the two groups. Unfortunately, the same outcome was achieved when propensity-score-matched comparisons were attempted to investigate the effect of dose escalation on OS stratified by histologic type and IMRT use.This was a retrospective database-based study, and conclusions were limited by lacking radiotherapy technique and planning, staging and chemotherapy regimens, and salvage regimens for recurrence and metastasis. In another study,^26^ twelve patients were assigned to receive a radiation dose of 61.2 Gy and 30 patients to receive 50.4 Gy; dose-related toxicities were encountered in two out of twelve patients in the high-dose group, including grade 3 esophagaomediastinal fistula and grade 4 pericardial effusion. However, it might be limited by the unbalanced sample size and the increased toxicity of chemoradiation therapy, which combines three cytotoxic agents.

However, several studies showed that higher radiation doses resulted in effective local control of locally advanced esophageal cancer. Suh et al^17^suggested that dose escalation in stage II–III locally advanced esophageal carcinoma improved the 2-year local control rate (69% versus 32%, P < .01) and PFS (47% versus 20%, P = .01), with no increase in treatment-related toxicity. Squamous cell carcinoma and adenocarcinoma were among the patients. Finally, high-dose radiotherapy of 60 Gy or more combined with concurrent chemotherapy is an effective therapeutic option for Stage II–III esophageal cancer. In another study,^16^ 44 patients with squamous cell carcinoma were divided into two groups, with the high-dose group receiving 63.9 Gy (boosted in primary lesion) and the low-dose group receiving 60 Gy, boosted high dose can increase relapse-free survival. Furthermore, He et al^27^included patients who were treated with doses of 50.4 Gy or more; HD-RT group had a considerably reduced local failure rate (17.9% versus 34.3%, p = .024) and a somewhat higher 5-year local-regional failure-free survival (68.7% versus 55.9%, p = .052) than the low-dose group.

More encouragingly, dose escalation will be a viable option for improving OS rather than just local control. Chen et al analyzed the results of 691 patients with esophageal squamous cell carcinoma after matching the general clinical data, HR of death was 0.75 (95% CI 0.64–0.88) when high-dose compared to standard dose. It concluded that higher radiotherapy dose led to better survival for locally advanced squamous cell carcinoma. Similarly, Kim et al^19^ retrospectively analyzed the clinical data of 236 patients with stage II–III esophageal cancer and compared 120 patients with radiotherapy doses <60 Gy to 116 patients with ≥60 Gy. The results showed that there was a significant local control advantage (69.1% versus 50.3%, P = .002) and survival advantage (35.1 months versus 22.3 months, P = .043) in high doses and the incidence of treatment-related adverse reactions was similar. In another study,^21^ higher doses than that used for standard radiotherapy resulted in higher 5-year OS rates (42.8% versus 21%). Similar findings were in other studies.^22–24^ According to the mentioned research, the benefit of OS and LC in the HD-RT group may base on the biological behavior of solid tumors and squamous cell carcinomas^28,29^and attributed to advancements in radiotherapy technology and imaging technology, which allow for more precise target delineation.And the selection bias of retrospective studies cannot be ignored.

Undoubtedly, the findings of three recent randomized controlled trials must be discussed. This study included the ARTDECO study^14^ and the study conducted by Xu et al,^15^ whereas the CONCORDE study^30^only reported the results of conference abstracts, which could not be included in this study. According to the ARTDECO study, there was no significant difference in PFS, OS, and Locoregional progression-free survival (LRPFS) between the HD-RT (61.6 Gy) group and LD-RT (50.4 Gy) group. The results may be related to the fact that dose escalation was only delivered to the primary tumor, and 39% of adenocarcinoma patients show different risk factors and biological characteristics from squamous carcinoma. In addition, an excess of fatal bleeding in grade 4 and 5 toxicities in the HD arm occurred. Similarly, Xu et al found no difference toward 3y-OS or PFS or LRPFS between the HD-RT (60 Gy) and LD-RT (50 Gy) groups, but grade 3+ radiation pneumonitis (7.5% versus 3.1%, nominal P = .03) increased. It should be noted that there was a difference in the RT completion rates (88.2% versus 96.9%, P < .01), poor implementation of the PET/CT scan may result in the improper stage, and a dose of 60 Gy may still be insufficient for LAEC, particularly for ESCC. Correspondingly, the results of the CONCORDE study reported at the 2021 American Society for Radiation Oncology (ASTRO) annual meeting showed no statistically significant difference in the primary endpoint 2y-LRPFS (43.8% versus 42.7%, p = .88) between the HD-RT (66 Gy) and LD-RT (50 Gy) groups. And this outcome could be attributed to 3D conformal radiotherapy technology. Conclusions cannot be drawn before the publication of the detailed data. Simultaneously, we should note that these three studies used radiotherapy with elective lymph nodal irradiation. And there is no discernible difference in treatment failure mode compared to previous involved field irradiation reports. The ESO-Shanghai 1 study^31^also found that involved field irradiation (IFI) resulted in lower irradiation toxicities without sacrificing OS in patients with LAEC. The preliminary findings from a multicenter, phase III clinical trial (NROG 001-Northern Radiation Oncology Group of China)^32^also supported this claim, with a better PFS and LRRFS in the high-dose IFI group. The final result lives up to our expectations. We are also looking forward to the SCOPE2 study(NCT 02741856),^33^ which will compare the effects of conventional dosages (50 Gy) to higher doses (60 Gy), as well as the impact of alternative chemotherapy regimens in patients who do not respond to standard drugs early in treatment. Furthermore, the ongoing NCT02556762 study, which compares 66 Gy(SIB) to 50 Gy, may determine whether higher doses than 60 Gy is beneficial.

Given the contradictory findings of the above studies, we conducted this meta-analysis and indicated that higher doses can benefit patients with LAEC in survival.Inevitably, there are some limitations in this study. At first, the quality of the included studies varied, with four retrospective studies. Second, a small patient population enrolled in some studies, including two RCTs. The studies conducted by Zhu et al and Nayan et al found no lung or esophageal side effects (grade≥3), which could be attributed to the small sample size and individual differences in radiation sensitivity. Another reason that Zhu et al. chose patients with neck and upper thoracic esophageal cancer was doubtless.Third, the stages of patients were based on different versions instead of individual patient data. Finally, some studies lacked critical information such as radiation field, dosage distribution, and chemotherapy regimens. These limitations may have an impact on the actual value of our research. More RCTs are needed to back up our findings.

## Conclusion

High-dose radiotherapy in definitive CCRT for patients with locally advanced esophageal carcinoma is associated with improved PFS, OS, cCR, and LC with no increase of grade≥3AEs. Simultaneously, we await the preliminary and final results of several ongoing dose-escalation randomized trials. Furthermore, future studies should provide personalized radiotherapy doses for these patients.

## Supplementary Material

Supplemental Material

## Data Availability

All data, models, and code generated or used during the study appear in the submitted article.

## References

[1] Siegel RL, Miller KD, Fuchs HE, Jemal A. Cancer statistics, 2022. CA Cancer J Clin. 2022;72(1):7–33. doi:10.3322/caac.21708. PMID: 35020204. 35020204

[2] Cooper JS, Guo MD, Herskovic A, Macdonald JS, Martenson JA Jr., Al-Sarraf M, Byhardt R, Russell AH, Beitler JJ, Spencer S, et al. Chemoradiotherapy of locally advanced esophageal cancer: long-term follow-up of a prospective randomized trial (RTOG 85-01). Radiation Therapy Oncology Group. JAMA. 1999;281:1623–1627. PMID: 10235156. doi:10.1001/jama.281.12.1623. 10235156

[3] DA AJA, BD TA, Chao J, Corvera C. National comprehensive cancer network. NCCN Clinical Practice Guidelines in Oncology (NCCN Guidelines).Esophageal and Esophagogastric Junction Cancers. Version 1 (2022). https://www.nccn.:org/professionals/physician_gls/pdf/esophageal.

[4] Minsky BD, Neuberg D, NKelsen DP, Kelsen DP, Pisansky TM, Ginsberg RJ, Pajak T, Salter M, Benson AB. Final report of Intergroup Trial 0122(ECOG PE-289, RTOG 90-12): phase II Trial of neoadjuvant chemotherapy plus concurrent chemotherapy and high-dose radiation for squamous cell carcinoma of the esophageal. Int J Radiat Oncol Biol Phys. 1999;43(3):517–523. doi:10.1016/s0360-3016(98)00463-5. PMID:10078631. 10078631

[5] Minsky BD, Pajak TF, Ginsberg RJ, Pisansky TM, Martenson J, Komaki R, Okawara G, Rosenthal SA, Kelsen DP. INT 0123 (radiation therapy oncology group 94-05) Phase III trial of combined-modality therapy for esophageal cancer: high-dose versus standard-dose radiation therapy. J Clin Oncol. 2002;20(5):1167–1174. doi:10.1200/JCO.2002.20.5.1167. PMID: 11870157. 11870157

[6] Welsh J, Settle S, Amini A, Amini A, Xiao L, Suzuki A, Hayashi Y, Hofstetter W, Komaki R, Liao Z, Ajani JA. Failure patterns in patients with esophageal cancer treated with definitive chemotherapy. Cancer. 2012;118(10):2632–2640. doi:10.1002/cncr.26586. PMID: 22565611. 22565611 PMC3747650

[7] Zhang Z, Liao Z, Jin J, Ajani J, Chang JY, Jeter M, Guerrero T, Stevens CW, Swisher S, Ho L, et al. Dose-response relationship in locoregional control for patients with stage II-III esophageal cancer treated with concurrent chemotherapy and radiotherapy. Int J Radiat Oncol Biol Phys. 2005;61(3):656–664. doi:10.1016/j.ijrobp.2004.06.022. PMID: 15708243. 15708243

[8] Li M, Zhang X, Zhao F, Luo Y, Kong L, Yu J. Involved-field radiotherapy for esophageal squamous cell carcinoma: theory and practice. Radiat Oncol. 2016;11(1):18. doi:10.1186/s13014-016-0589-7. PMID: 26846932. 26846932 PMC4743321

[9] Song T, Liang X, Fang M, Wu S. High-dose versus conventional-dose irradiation in cisplatin-based definitive concurrent chemoradiotherapy for esophageal cancer: a systematic review and pooled analysis. Expert Rev Anticancer Ther. 2015;15(10):1157–1169. doi:10.1586/14737140.2015.1074041. PMID: 26235427. 26235427

[10] Chen Y, Zhu HP, Wang T, Sun C-J, Ge X-L, Min L-F, Zhang X-W, Jia -Q-Q, Yu J, Yang J-Q. What is the optimal radiation dose for non-operable esophageal cancer? Dissecting the evidence in a meta-analysis. Oncotarget. 2017 Jun 28;8(51):89095–89107. 10.18632/oncotarget.18760. PMID: 29179502. 29179502 PMC5687672

[11] Luo HS, Huang HC, Lin LX. Effect of modern high-dose versus standard-dose radiation in definitive concurrent chemo-radiotherapy on outcome of esophageal squamous cell cancer: a meta-analysis. Radiat Oncol. 2019;14(1):178. doi:10.1186/s13014-019-1386-x. PMID: 31623639. 31623639 PMC6798457

[12] Sun X, Wang L, Wang Y, Kang J, Jiang W, Men Y, Hui Z. High vs. Low radiation dose of concurrent chemoradiotherapy for esophageal carcinoma with modern radiotherapy Techniques: a meta-analysis. Front Oncol. 2020;10:1222. doi:10.3389/fonc.2020.01222. PMID: 32850362. 32850362 PMC7418493

[13] Xiao LL, Czito B, Wang J, Jing S. Do higher radiation doses with concurrent chemotherapy in the definitive treatment of esophageal cancer improve outcomes? A meta-analysis and systematic review. International Journal of Radiation Oncology Biology Physics. 2020;108(3):e611. doi:10.1016/j.ijrobp.2020.07.1856. PMID: 32489478. PMC7255355 32489478

[14] Hulshof MCCM, Geijsen D, Rozema T, Oppedijk V, Buijsen J, Neelis KJ, Nuyttens J, Van Der Sangen M, Jeene P, Reinders J, et al. A randomized controlled phase III multicenter study on dose escalation in definitive chemoradiation for patients with locally advanced esophageal cancer: ARTDECO study. J Clin Oncol. 2020 2020 Jun 8;39(25):2816–2824. 10.1200/JCO.20.03697. PMID: 34101496. 34101496

[15] Xu Y, Dong B, Zhu W, Li J, Huang R, Sun Z, Yang X, Liu L, He H, Liao Z, et al. Phase III Multicenter Randomized Clinical Trial of 60 Gy versus 50 Gy Radiation Dose in Concurrent Chemoradiotherapy for Inoperable Esophageal Squamous Cell Carcinoma. Clin Cancer Res. 2022; 28:1792–1799. 10.1158/1078-0432.Ccr-21-3843. PMID: 35190815. 35190815

[16] Zhu WG, Zhou K, Yu CH, Han JH, Li T, Chen XF. Efficacy analysis of simplified intensity-modulated radiotherapy with high or conventional dose and concurrent chemotherapy for patients with neck and upper thoracic esophageal carcinoma. Asian Pac J Cancer Prev. 2012;13(3):803–807. doi:10.7314/apjcp.2012.13.3.803. PMID: 22631652. 22631652

[17] Suh Y-G, Lee IJ, Koom WS, Cha J, Lee JY, Kim SK, Lee CG. High-dose versus standard-dose radiotherapy with concurrent chemotherapy in Stages II–III esophageal cancer. Japanese Journal of Clinical Oncology. 2014 Jun;44(6):534–540. 10.1093/jjco/hyu047. PMID: 24771865. 24771865

[18] Chen CY, Li CC, Chien CR. Does higher radiation dose lead to better outcome for non-operated localized esophageal squamous cell carcinoma patients who received concurrent chemoradiotherapy? A population based propensity-score matched analysis. Radiother Oncol. 2016;120(1):136–139. doi:10.1016/j.radonc.2016.04.042. PMID: 27207358. 27207358

[19] Kim HJ, Suh Y-G, Lee YC, Lee SK, Shin SK, Cho BC, Lee CG. Dose-Response relationship between radiation dose and loco-regional control in patients with Stage II-III esophageal cancer treated with definitive chemoradiotherapy. Cancer Res Treatment. 2017 Jul;49(3):669–677. 10.4143/crt.2016.354. PMID: 27737537. PMC5512369 27737537

[20] Nayan N, Bhattacharyya M, Jagtap VK, Kalita AK, Sunku R, Roy PS. Standard-dose versus high-dose radiotherapy with concurrent chemotherapy in esophageal cancer: a prospective randomized study. South Asian Journal of Cancer. 2018;7(1):27–30. doi:10.4103/sajc.sajc_178_17. PMID: 29600230. 29600230 PMC5865091

[21] Zhang W, Luo Y, Wang X, Han G, Wang P, Yuan W, Dai SB. Dose-escalated radiotherapy improved survival for esophageal cancer patients with a clinical complete response after standard-dose radiotherapy with concurrent chemotherapy. Cancer Manag Res. 2018;10:2675–2682. PMID: 30147366. doi:10.2147/cmar.S160909. 30147366 PMC6097517

[22] Li CC, Fang HY, Lin CY, Shen WC, Chien CR. Outcomes of localized esophageal squamous cell carcinoma patients treated with definitive concurrent chemoradiotherapy using either standard or high radiotherapy dose: a retrospective study controlling for organ at risk dose. Anticancer Research. 2019;39(1):511–517. doi:10.21873/anticanres.13142. PMID: 30591503. 30591503

[23] Li CC, Chen CY, Chou YH, Huang CJ, Ku HY, Chien CR. Optimal radiotherapy dose in cervical esophageal squamous cell carcinoma patients treated with definitive concurrent chemoradiotherapy: a population based study. Thoracic Cancer. 2021;12(14):2065–2071. doi:10.1111/1759-7714.14009. PMID: 34028200. 34028200 PMC8287021

[24] Zhu H, Lu X, Jiang J, Lu J, Sun X, Zuo Y. Radiotherapy combined with concurrent nedaplatin-based chemotherapy for Stage II-III esophageal squamous cell carcinoma. Dose-response: a Publication of International Hormesis Society. 2022;20(1):15593258221076720. doi:10.1177/15593258221076720. PMID: 35273471. 35273471 PMC8902195

[25] Brower JV, Chen S, Bassetti MF, Yu M, Harari PM, Ritter MA, Baschnagel AM. Radiation dose escalation in esophageal cancer revisited: a contemporary analysis of the national cancer data base, 2004 to 2012. Int J Radiat Oncol Biol Phys. 2016;96(5):985–993. doi:10.1016/j.ijrobp.2016.08.016. PMID: 27869098. 27869098

[26] Higuchi K, Komori S, Tanabe S, Katada C, Azuma M, Ishiyama H, Sasaki T, Ishido K, Katada N, Hayakawa K, et al. Definitive chemoradiation therapy with docetaxel, cisplatin, and 5-fluorouracil (DCF-R) in advanced esophageal cancer: a phase 2 trial (KDOG 0501-P2). Int J Radiat Oncol Biol Phys. 2014;89(4):872–879. doi:10.1016/j.ijrobp.2014.03.030. PMID: 24867539. 24867539

[27] He L, Allen PK, Potter A, Wang J, Chang JY, Gomez DR, Komaki R, Liao Z, Lin SH. Re-evaluating the optimal radiation dose for definitive chemoradiotherapy for esophageal squamous cell carcinoma. J Thorac Oncol. 2014;9(9):1398–1405. doi:10.1097/jto.0000000000000267. PMID: 25122435. 25122435

[28] GH F. clinical dose response curves of human malignant epithelial tumors. Br J Radiol. 1973;46:151. 10.1259/0007-1285-46-541-1. PMID: 4630323. 4686826

[29] Siewert JR, O K. Are squamous and adenocarcinomas of the esophagus the same disease? Semin Radiat Oncol. 2007;17(1):138–144. doi:10.1016/j.semradonc.2006.09.007. PMID: 17185196. 17185196

[30] Crehange G, M’vondo C, B A. 2021. Exclusive chemoradiotherapy with or without radiation dose escalation in esophageal cancer: multicenter phase 2/3 randomized trial CONCORDE(PRODIGE-26). Int J Radiat Oncol Biol Phys. 111(3):S5. 10.1016/j.ijrobp.2021.07.045.

[31] Zhu H, Rivin Del Campo E, Y J, Simone CB, Zhu Z, Zhao W, Amini A, Zhou J, Wu C, Tang H. Involved-field irradiation in definitive chemoradiotherapy for locoregional esophageal squamous cell carcinoma: results from the ESO-Shanghai 1 trial. Int J Radiat Oncol Biol Phys. 2021;110(5):1396–1406. doi:10.1016/j.ijrobp.2021.02.053. PMID: 33677048. 33677048

[32] Li B, Zhang J, Zhang K, Li G. 2019. Chemoradiation with ENI versus IFI, high-dose versus standard-dose radiation therapy for locally advanced esophageal squamous cell carcinoma: preliminary results of multicenter, Phase III clinical trial (NROG 001-Northern radiation oncology group of China). Int J Radiat Oncol Biol Phys. 105(Supplement):E188–9. 10.1016/j.ijrobp.2019.06.2096.

[33] G S, H E, PK A. Driving developments in UK oesophageal radiotherapy through the SCOPE trials. Radiat Oncol. 2019;14(1):26. doi:10.1186/s13014-019-1225-0. PMID: 30717810. 30717810 PMC6360789

