# Supplementary material for: The impact of radiation dose on the efficacy of definitive chemoradiotherapy in patients with locally advanced esophageal carcinoma: a systematic review and meta-analysis
Source: Cancer Biol Ther. 2022 Dec 15;24(1):1–10. doi: 10.1080/15384047.2022.2156246 (PMC9762832; doi:10.1080/15384047.2022.2156246)
Supplement: Supplemental Material [file KCBT_A_2156246_SM1907.docx]

Figure 1 Caption: Flow chart of studies selection procedure.

Figure 1 Alt Text: Eleven studies met the criteria and were incorporated into the meta-analysis

Figure 2 Caption: Forest plots for relationship between HD-RT and LD-RT.(A) pooled analyses for OS;(B)pooled analyses for PFS.

Figure 2A Alt Text: All articles reported overall survival in groups. Patients in the HD-RT group had significant survival benefits compared to patients in the LD-RT group (pooled HR 0.78 [0.70, 0.87], p＜0.00001).

Figure 2B Alt Text: Five studies analyzed the PFS of the two groups. HD-RT group had a significant advantage over LD-RT group (HR 0.72 [0.55, 0.94], p=0.01).

Figure 3 Caption: Forest plot for relationship between HD-RT and LD-RT.(A) odds ratio for cCR;(B) odds ratio for LRF;(C) odds ratio for DM.

Figure 3A Alt Text: Six studies compared the cCR rates of the two groups, with the HD-RT group outperforming the LD-RT group in cCR (OR 1.52 [1.13, 2.05]; P=0.005).

Figure 3B Alt Text: Six studies compared the LRF rates of the two groups, with the HD-RT group outperforming the LD-RT group in LRF (OR 0.60 [0.45, 0.80], p=0.0004).

Figure 3C Alt Text: Five articles analyzed the DM rates of the two groups. There was no difference between the two groups in this respect (OR 1.43 [1.00, 2.04]; P=0.05).

Figure 4 Caption: Effect of HD-RT and LD-RT on grade≥3 AEs.Odds ratio for (A)pneumonitis;

(B)esphagitis;(C)other esophageal toxicities:stenosis/fistula/hemorrhage;(D)treatment-related death.

Figure 4A Alt Text: No significant difference was demonstrated between the two arms in terms of grade 3–5 radiation pneumonitis (OR 1.38 [0.71, 2.68], p=0.35).

Figure 4B Alt Text: No significant difference was demonstrated between the two arms in terms of grade 3–5 radiation esophagitis (OR 1.36 [0.88, 2.10], p=0.17).

Figure 4C Alt Text: No significant difference was demonstrated between the two arms in terms of grade 3–5 radiation Other Esophageal toxicities (stenosis/ fistula/ hemorrhage) (OR 1.22 [0.75, 2.00], p=0.43).

Figure 4D Alt Text: No significant difference was demonstrated between the two arms in terms of treatment-related death (OR 1.40 [0.73, 2.68], p=0.31).

Figure 5 Caption: Sensitivity analyses for (A) OS and (B) PFS.Publication bias funnel plot: Beggs test and Eggers test for (C and D) OS and (E and F) PFS.

Figure 5A Alt Text: Sensitivity analysis revealed that the new HRs for OS was identical to the original HRs, demonstrating that no single study may have significantly influenced the meta-analysis results.

Figure 5B Alt Text: Sensitivity analysis revealed that the new HRs for PFS was identical to the original HRs, demonstrating that no single study may have significantly influenced the meta-analysis results.

Figure 5C Alt Text: No significant publication bias was found between HD-RT group and LD-RT group among all studies on OS (Begg's test, P= 0.533).

Figure 5D Alt Text: No significant publication bias was found between HD-RT group and LD-RT group among all studies on OS (Egger's test, p=0.325[ -2.6350,0.9731]).

Figure 5E Alt Text: No significant publication bias was found between HD-RT group and LD-RT group on PFS (Begg's test, P= 0.086).

Figure 5F Alt Text: No significant publication bias was found between HD-RT group and LD-RT group on PFS (Egger's test, p= 0.148[ -9.7395, 2.3638]).
